# Eating Too Much

**Published:** 1855-10

**Authors:** 


### EATING TOO MUCH.

What countless thousands it puts into the doctor's pockets, furnishes his splendid mansion in Union Square and Fifth Avenue, enables him to "sport his carriage," to own a villa on the banks of the Hudson, and live in style to the end of the chapter!

"*I can't help it*," says the poor unfortunate milk-and-water individual, who never had decision enough to do a deed worthy of remembrance an hour later. My wishey-washey friend, suppose I help you to avoid making a beast of yourself.

Have two articles of food sent to your room, besides bread and butter, with half a glass of cold water. I will give you permission to eat as much as you want, thus, thrice a day. Or if you prefer eating with company, you may safely sit down to the "*best table*" in the land, if you have manhood enough to partake of but any two articles. *It is the variety of our food which brutifies us.*

